# Aqueous Ethanolic Extract of *Tinospora cordifolia* as a Potential Candidate for Differentiation Based Therapy of Glioblastomas 

**DOI:** 10.1371/journal.pone.0078764

**Published:** 2013-10-24

**Authors:** Rachana Mishra, Gurcharan Kaur

**Affiliations:** Department of Biotechnology, Guru Nanak Dev University, Amritsar, India; University of Pécs Medical School, Hungary

## Abstract

Glioblastomas are the most aggressive primary brain tumors and their heterogeneity and complexity often renders them non responsive to various conventional treatments. Search for herbal products having potential anti-cancer activity is an active area of research in the Indian traditional system of medicine i.e., Ayurveda. *Tinospora cordifolia*, also named as ‘heavenly elixir’ is used in various ayurvedic decoctions as panacea to treat several body ailments. The current study investigated the anti-brain cancer potential of 50% ethanolic extract of *Tinospora cordifolia* (TCE) using C6 glioma cells. TCE significantly reduced cell proliferation in dose-dependent manner and induced differentiation in C6 glioma cells, resulting in astrocyte-like morphology as indicated by phase contrast images, GFAP expression and process outgrowth data of TCE treated cells which exhibited higher number and longer processes than untreated cells. Reduced proliferation of cells was accompanied by enhanced expression of senescence marker, mortalin and its translocation from perinuclear to pancytoplasmic spaces. Further, TCE showed anti-migratory and anti-invasive potential as depicted by wound scratch assay and reduced expression of plasticity markers NCAM and PSA-NCAM along with MMP-2 and 9. On analysis of the cell cycle and apoptotic markers, TCE treatment was seen to arrest the C6 cells in G0/G1 and G2/M phase, suppressing expression of G1/S phase specific protein cyclin D1 and anti-apoptotic protein Bcl-xL, thus supporting its anti-proliferative and apoptosis inducing potential. Present study provides the first evidence for the presence of anti-proliferative, differentiation-inducing and anti-migratory/anti-metastatic potential of TCE in glioma cells and possible signaling pathways involved in its mode of action. Our primary data suggests that TCE and its active components may prove to be promising phytotherapeutic interventions in gliobalstoma multiformae.

## Introduction

Glioblastomas are the most aggressive and highly invasive primary brain tumor types. The complexity and highly dynamic nature of multiple oncogenic pathways involved in the survival of these tumor cells renders them nonresponsive to various available radiotherapeutic and chemotherapeutic treatments. Plants are the safest source of therapeutic agents, having multi-targeted mode of action with least or no side effects. *Tinospora cordifolia* is one of the most widely used rasayana herb in ayurveda and commonly known as ‘Giloy’, a mythological term, that refers to ‘heavenly elixir’ or ‘Amrita’. The plant extract is being used as an important component of various ayurvedic formulations that are used for improving general body health [1,2]. Various bioactive components have already been isolated from *Tinospora cordifolia* which belongs to different classes of compounds such as alkaloids, diterpenoid, lactones, glycosides, steroids, sesquiterpenoid, phenolics, aliphatic compounds and polysaccharides. This plant has been used as remedy for jaundice and its extracts and purified components have been found to have hepato-protective effect against various toxic agents [3,4]. The crude extract and various compounds isolated from this plant have been reported to have several medicinal properties [5] including immunomodulatory [6] and immunostimulatory activity [7,8] that helps in increasing immune response by the lymphocytic cells [9], macrophages [10] and dendritic cells [11]. Several recent reports have suggested that the plant extract is a rich source of biochemicals that have potential therapeutic value in treating diabetes and related disorders caused by disturbed carbohydrate metabolism [12–17]. Apart from this, many previous studies have provided evidence for the presence of adaptogenic [18], cardioprotective [19], antioxidant [20,21] anti-inflammatory [22,23], and antipsychotic [24] activities in this plant. Amazingly this plant shows radio-sensitizing activity in cancerous cells [25,26] but on the other hand protects normal cells from hazardous effects of radiations [27,28]. The plant extract and epoxy cleordane isolated from this plant have been shown to possess chemoprotective potential [29–31]. Several recent studies have reported that various extracts of *Tinospora cordifolia* plant possess bioactive components which inhibit cellular proliferation in various *in vitro* models and also show antineoplastic [32], antitumor [33–35], anti-angiogenesis [36,37] and anti metastatic activity in various *in vivo* models [35,37,38].

The present study was aimed to explore whether 50% ethanolic extract of *Tinospora cordifolia* (TCE) exhibits potential anti-proliferative, pro-apoptotic and anti-migratory activity along with differentiation and senescence inducing potential in glioma cells. *N-*nitrosomethyl-urea induced rat C6 glioma cell line has been a widely accepted model for study of glioblastoma growth and metastasis [39]. Oncogenesis and neoplasia in brain cells including glial cells involve altered signaling cascades related to differentiation, adhesion and apoptosis. Thus, we focussed our study on markers related to these pathways in C6 glioma cells following TCE treatment. Glial fibrillary acidic protein (GFAP), an intermediate filament protein, has been well established as a differentiation marker for glial cells in normal brain. Further expression of senescence marker i.e., mortalin, a highly conserved heat shock chaperon localized in different subcellular locations, was evaluated. Mortalin has been implicated in various functions ranging from stress response, control of cell proliferation, and inhibition/prevention of apoptosis [40–42], whereas, mitochondrial heat shock protein HSP70, also known as stress response protein, is well reported to play a vital role in critical differentiation and proliferation stages in cells during early mammalian development [43,44]. Thus, expression of HSP70 along with plasticity markers NCAM and its polysialylated form PSA-NCAM were evaluated to explore their role in TCE mediated inhibition of proliferation and rate of migration of C6 glioma cells.

NCAM, which belongs to the immunoglobulin superfamily, is involved in multiple neuronal interactions that influence cell migration, axonal and dendritic projection, and synaptic targeting. It is actively involved in the process of morphogenesis, neural cell differentiation, axonal outgrowth and fasciculation [45]. With its three isoforms i.e., 180, 140 and 120 kDa, NCAM is not only involved in mediating signaling pathways for neural development and plasticity but also in oncogenesis [46,47]. The spatio-temporal pattern of expression of PSA-NCAM, an important post translational modification of NCAM, is not only critical for proper neural morphogenesis but also reported to have important role in tumorigenesis and metastasis [48–50]. Since, differentiation is accompanied by cell cycle arrest and induction of apoptosis, we also studied anti-apoptotic protein family molecule bcl-xl along with cyclin D1 which is a proto-oncogene and involved in G1 to S phase transition in cell cycle. These anti-apoptotic molecules have been shown to play major role in cellular differentiation, apoptosis and cell cycle [51,52]. We provide first evidence that TCE exhibits anti-proliferative, pro-apoptotic and anti-migratory activity in C6 glioma cells and these effects were accompanied by induction of differentiation and senescence related pathways.

## Materials and Methods

The 50% ethanolic extract of *Tinospora cordifolia* stem (TCE) was obtained from Indian Institute of Integrative Medicine, Jammu, India. The air dried extract was reconstituted in 50% ethanol at 100 mg/ml concentration, which was further diluted in DMEM with 10% FBS according to experimental requirement. 

### Chemical standardization of TCE and nature of active component/s

TCE was subjected to preliminary phytochemical screening for alkaloids, amino acids, resins, flavonoids, phytosterols, saponins, steroids, tannins, terpenoids and reducing sugars following the methods of Harborne [53] and Kokate [54]. The dried 50% ethanolic extract was further fractionated with hexane, chloroform, ethyl acetate and butanol. All the fractions were then tested for bioactivity and bioactive fraction were further subfractionated on TLC plate. All the subfractions were then again tested for antiproliferative property.

### Cell culture and treatment

Rat C6 glioma, U87MG human glioma, PC3 prostate cancer cell line and HeLa cell line were obtained from National Centre for Cell Science (Pune, India). The cells were routinely grown in DMEM supplemented with 10% FBS (Biological Industries) and 1X PSN mix (Invitrogen) at 37°C in a humidified atmosphere containing 5% CO_2_. Cells were subcultured by trypsinization and seeded in 96 and 24 well plates according to the requirement of the experiments. At the confluency of 30-40%, cells were treated with TCE, ranging from concentration 10 μg/ml to 1000 μg/ml in 96 well plates before selection of final doses of 250 μg/ml and 350 μg/ml for further experiments. Cultures were incubated for 72 h.

### Proliferation assays

TCE was tested for anti-proliferative activity and cytotoxicity by MTT test on C6, U87MG, PC3 and HeLa cells using the 3-(4, 5-dimethylthiazol-2-yl)-2, 5- diphenyltetrazolium bromide (MTT) by measuring formation of formazan crystals by mitochondrial dehydrogenase [55]. 

### Cellular and nuclear morphology studies

Morphological changes in glioma cells treated with different concentrations of TCE were imaged with phase contrast microscopy and nuclear morphology was studied by staining with DAPI stain (4', 6-diamidino-2-phenylindole) a fluorescent stain that specifically binds to AT rich region of DNA.

### Process outgrowth analysis

In order to explore differentiation inducing potential of TCE, C6 cells were studied for number and length of process outgrowths. C6 cells were seeded in 12 well plates. After incubation with TCE, cells were fixed with 2.5% of glutaraldehyde for 90 min followed by washing with PBS and staining with staining solution containing 1% toluidine blue and 1% methylene blue in 1% sodium tetra borate for 1 h. Cells were then washed with water and kept for drying at room temperature. Cells were photographed with Nikon Cool Snap CCD camera. 100 cells each from control and TCE treated groups were analysed for number and length of processes using Image Pro Plus software version 4.5.1 from media cybernetics. 

### Immunostaining

Both control and treated cells were fixed with acetone and methanol (1:1) followed by permeabilization with 0.3% Triton-X 100 in phosphate buffered saline (0.3% PBST). Cells were incubated with mouse monoclonal anti-GFAP (1:500), anti-mortalin (1:500), anti-HSP70 (1:500), anti-cyclin D1 (1:250), anti-bcl-xl (1:200), anti-NCAM (1:500) all from Sigma and anti-PSA-NCAM (1:250) from Millipore diluted in 0.1% PBST, for 24 h at 4°C in humid chamber. For anti-PSA-NCAM staining, no permeabilization was done. Secondary antibody (goat anti-mouse IgG/IgM Alexaflour 488/543 from Invitrogen) was applied for 2 h at room temperature. Cells were then mounted with anti-fading reagent (Sigma) and images were captured by Nikon A1R Confocal Laser Microscope and the pictures were analyzed using NIS elements AR analysis software version 4.11.00.

### Protein assay and Western blotting

C6 glioma cells, grown and treated in 100 mm petri dishes, were harvested with PBS–EDTA (1 mM). Cell pellet was homogenized in RIPA buffer (50 mM Tris (pH 7.5), 150 mM NaCl, 0.5% sodium deoxycholate, 0.1% SDS, 1.0% NP-40) and protein content in the supernatant was determined by the Bradford method. Protein lysate (20–30 μg) was resolved in 10% and 7% gels by SDS-PAGE, followed by blot transfer onto a PVDF membrane (Hybond-P) using the semidry Novablot system (Amersham Pharmacia). Further, membranes were probed with mouse monoclonal anti-GFAP (1:3000), anti-mortalin (1:1000), anti-HSP70 (1:2500), anti-NCAM (1:2000) or anti-PSA-NCAM (1:1000), anti-bcl-xl (1:1000) and anti-cyclin D1 (1:2000) antibodies for overnight at 4°C. Membranes were then washed 3 times with 0.1% TBST for 15 min each and then incubated with HRP labelled anti mouse secondary antibody for 2 h. Immunoreactive bands were detected by ECL Plus Western blot detection system (Amersham Biosciences) using LAS 4000 (GE Biosciences). To rule out the possibility for potential variations in protein estimation and sample loading, expression of α-tubulin (endogenous control) was analysed on the same membrane after stripping and reprobing with anti-*α*-tubulin antibody. Final expression of each protein was calculated by normalising the expression of that protein by expression of α-tubulin in the same sample. 

### mRNA expression by quantitative Real Time PCR assay

Total RNA was extracted from the cells by TRI reagent (Sigma) according to manufacturer’s instructions. Equal amount of RNA was used for cDNA synthesis. A reaction volume of 20 μl for cDNA synthesis containing 200 U of M-MLV reverse transcriptase, 4 μl 5X first strand buffer, 2 μl of 1 M DTT, 5 μg of RNA, 20 U of ribonulease inhibitor, 250 ng pd (N_6_) random hexamer (Invitrogen), and 1 mM each of dNTPs (Amersham). 100 ng of cDNA was amplified in 10 μl of reaction mixture containing 5 μl of 2X TaqMan Master Mix, 0.5 μl of 20X predesigned TaqMan Primer Probe mix (Applied Biosystem). All reactions were performed in triplicate on StepOne Plus Real Time PCR system (Applied Biosystem). Amplification conditions comprised of initial holding stage of 50°C for 2 min after that 95°C for 10 min, and then cycling stage comprised of 40 cycles of amplification (denaturation at 95°C for 15 sec, further annealing and elongation at 60°C for 1 min). For each gene of interest, 18S ribosomal RNA was used as endogenous control. The value of each Ct was normalized by Ct value of 18S ribosomal RNA. The relative gene expression of each gene was defined as 2^-ΔΔCt^ and final gene expression was represented as 2^-ΔΔCt±SEM^.

### Annexin-V-FITC study for apoptosis

To determine whether TCE causes apoptotic and necrotic cell death, cells were stained with annexin V conjugated with FITC and PI using the annexin V-FITC apoptosis Detection Kit (Miltenyi Biotech), according to the manufacturer’s protocol. Annexin V has a high affinity for phosphatidylserine exposed on the outer membrane of apoptotic cells, while PI is transported to late-stage apoptotic/necrotic cells with disrupted cell membranes. The cells from control and treated groups were trypsinized, washed with PBS, and resuspended in 1ml of annexin V binding buffer (1X) with addition of 10 μl annexin V-FITC. Following incubation (for 15 min in the dark at room temperature) and centrifugation (5 min, 300xg), 500 μl of annexin V binding buffer and 5 μl of PI were added to the cell pellet and incubated for further 5 min in the same conditions. Viable (annexin V-, PI-negative), early apoptotic (annexin V-positive, PI-negative), late apoptotic (annexin V-, PI-positive) and necrotic (annexin V-negative, PI-positive) cells were detected by flow cytometry (Accuri C6 flow cytometer; Becton–Dickinson) and quantified by BD Accuri software.

### Cell cycle analysis

Cells were seeded in 100 mm diameter petri plates at the cell density 2.5x10^5^ per ml and then grown either in the presence or absence of TCE. After incubation of 72 h, cells were trypsinized, collected along with floating cells and then centrifuged at 2000 rpm. Cell pellet was resuspended in 1 ml of ice-cold PBS and then fixed with ice-cold 70% ethanol. Cells were centrifuged and resuspended in 1 ml of PBS and incubated for 15 min and centrifuged and resuspended in PI staining solution (100 mM Tris pH 7.4, 150 mM CaCl_2_, 0.5 mM MgCl_2_, 0.1% NP-40 and 3 μM PI) and studied with BD Accuri C6 Flow cytometer (BD Biosciences). DNA content histograms and cell cycle phase distribution were modelled from at least 50,000 single events by excluding cell aggregates based on scatter plots of Fluorescence pulse area versus fluorescence pulse width using FCS Express 4 flow research edition software (De novo software). 

### Wound scratch assay

In order to investigate anti-migration potential of TCE, C6 cells were grown to confluent monolayer. Monolayer was wounded by scratching the surface with a needle. Following the treatment with TCE, the initial wounding and the movement of cells in the scratched area were photographically monitored for 6 h after the treatment. Images were analysed by Image Pro Plus software version 4.5.1 from the media cybernetics.

### Gelatin zymogram study

In order to study the effect of TCE on Matrix Metalloproteinase, samples of supernatant medium conditioned by cell culture under different experimental conditions were separated on a 10% SDS-PAGE containing 0.1% gelatin. After electrophoresis, gels were washed with 2.5% Triton X-100 (in 50 mM Tris-HCl) for 30 min to remove SDS, followed by incubating the gel in zymogram developing buffer (Invitrogen) at 37°C for 48 h. Gels were subsequently stained with Coomassie brilliant blue and destained in buffer containing 50% methanol and 10% acetic acid (v/v), and the location of gelatinolytic activity was detected as clear bands.

### Statistical analysis

Values were expressed as mean ± SEM. The Sigma Stat for Windows (version 3.5) was adopted to analyze the results by Student’s *t*-test and one way ANOVA, in order to determine the significance of the means. Values of *P*<0.05 were considered as statistically significant.

## Results

### TCE impeded proliferation rate and induced differentiation in cancer cells

**Figure 1:**
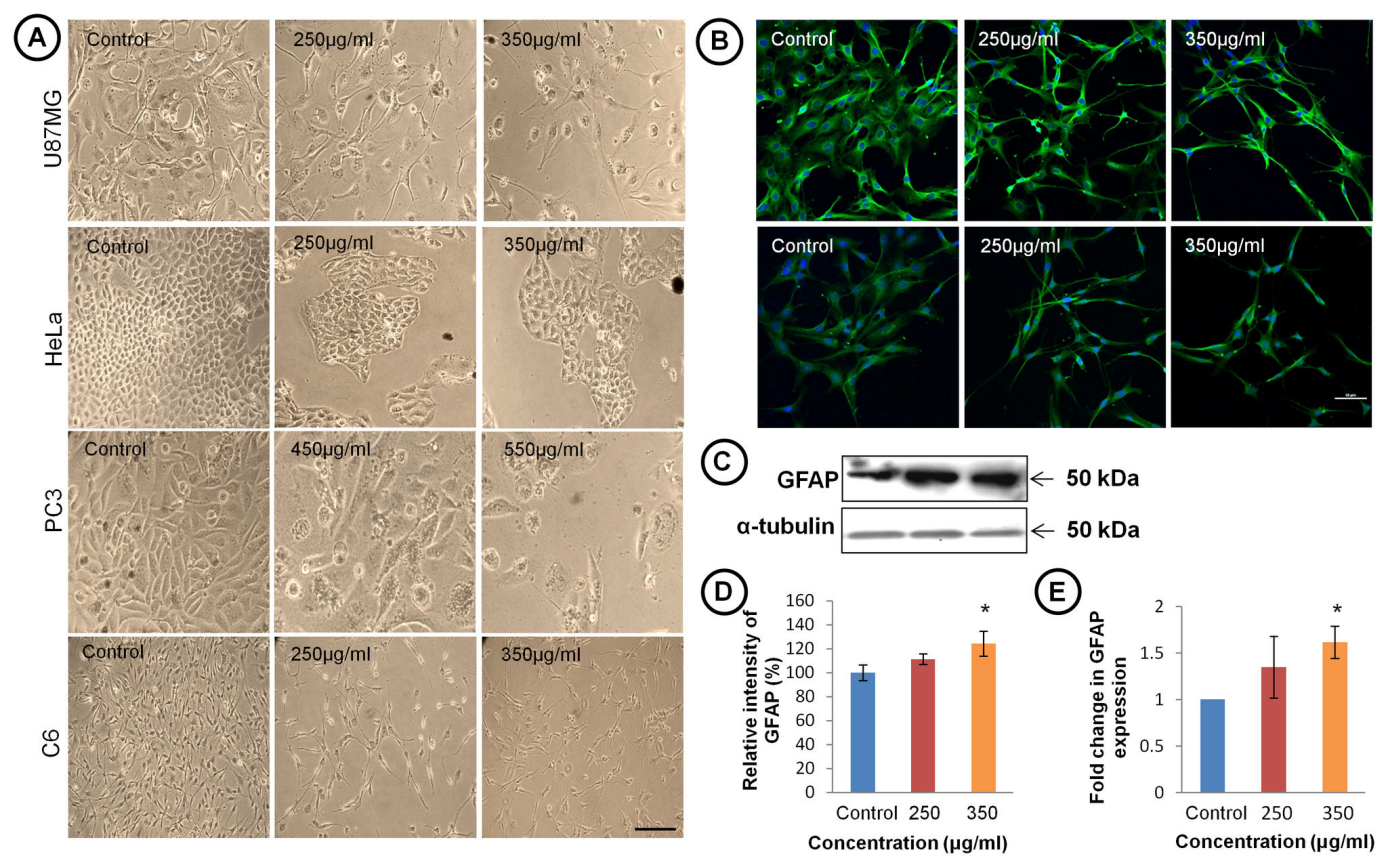
TCE Induces differentiation in U87MG, HeLa, PC3 and C6 cells. (**A**) Phase contrast photomicrographs of U87MG, HeLa, PC3 and C6 cell lines treated with TCE showing gradual changes from undifferentiated to highly differentiated morphology. Scale bar- 200 μm. (**B**) Confocal images of C6 glioma cells showing α-tubulin (upper panel) and GFAP (lower panel) expression. Scale bar- 50 μm. (**C**) Representative western blot hybridization signals of GFAP expression. (**D**) Histogram showing densitometric analysis of GFAP protein bands in western blotting in TCE treated and control groups. (**E**) Histograms representing mRNA expression of GFAP in control and treated groups. Gene expression is represented by ΔΔCt value of GFAP after normalising with 18S RNA as endogenous control. Values are presented as mean ± SEM of at least three independent experiments. ‘*’ (*P*<0.05) and ‘**’ (*p*< 0.01) represent statistical significant difference between control and TCE treated groups.

Phase contrast photomicrograph of C6, U87MG, HeLa and PC3 cell lines cultured in the presence of different concentrations of TCE (100-350 μg/ml) appeared to be growth arrested and showed morphology similar to normal differentiated cells with multiple and elongated processes. Differentiation was observed at slightly higher concentration (≥450 μg/ml) in case of PC3 cells. C6, U87MG and HeLa cells showed no cytotoxicity even at higher concentration (≥500 μg/ml of TCE) though the cell number was greatly reduced and cells appeared to be completely growth arrested (Figure 1A). Immunostaining with α-tubulin showed that TCE treated cells attained highly differentiated morphology with multiple and long stellate processes and small cell body. Induction of differentiation in C6 cells was further confirmed by the expression of GFAP, a differentiation marker for astrocyte cells, which was found to be enhanced significantly in TCE treated cells in comparison to untreated cells (Figure 1B). Western blot analysis and real time quantitative PCR data further supports that increase in expression of GFAP in TCE treated cells occurs both at translational as well as at transcriptional levels (Figure 1 D and E). 

**Figure 2:**
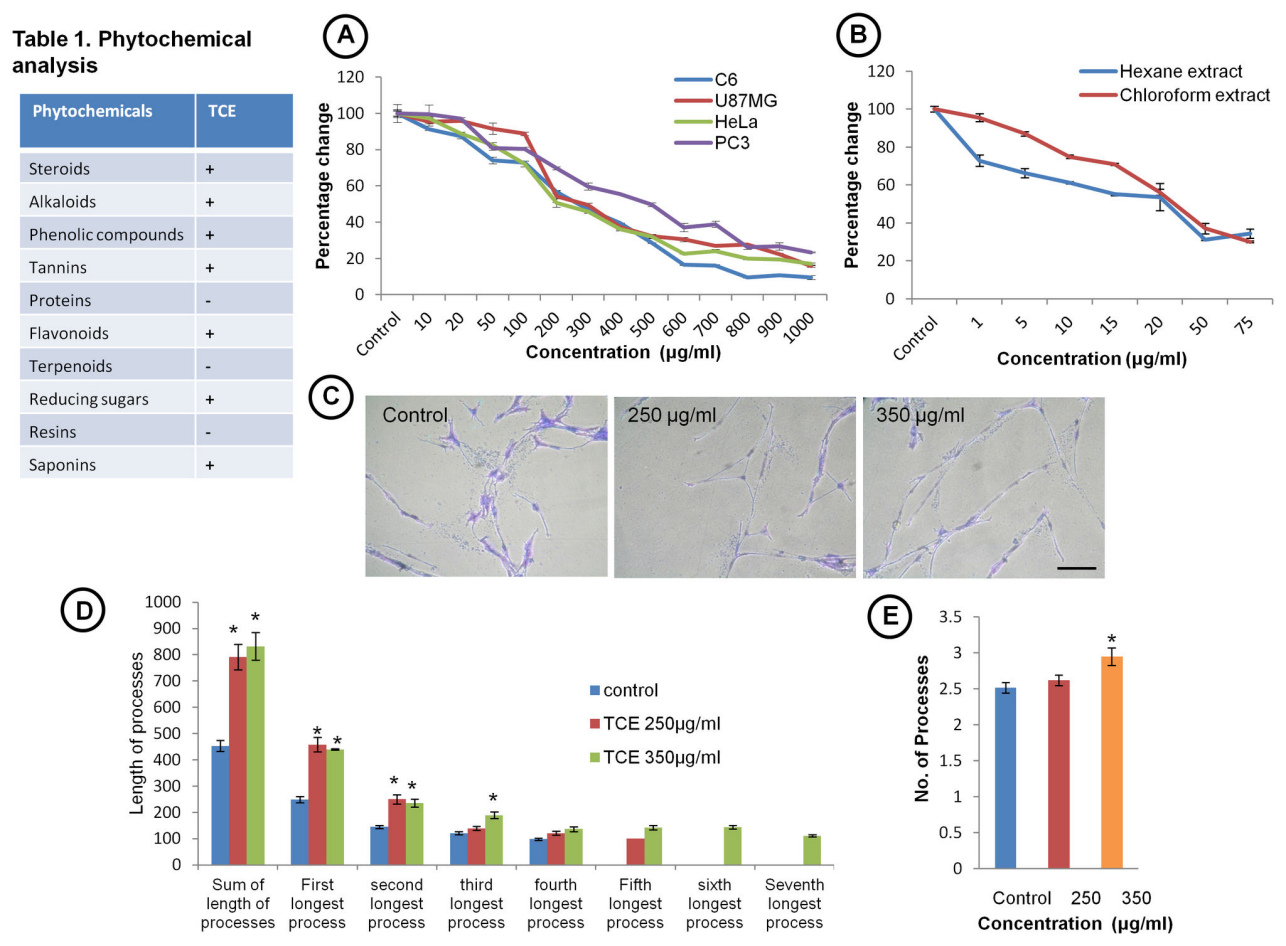
TCE treatment inhibits cell proliferation and induces process outgrowth. (Table 1) Phytochemical analysis of TCE. (**A**) MTT assay showing dose dependent decrease in cell number in TCE treated U87MG, HeLa, PC3 and C6 cells. Graph showing IC_50_ for U87MG, HeLa and C6 cells at 200 μg/ml and for PC3 cells at 500 μg/ml. (**B**) MTT assay showing the effect of hexane and chloroform fractions on C6 glioma cells. (**C**) Bright field images of cells stained with 1% toluidine blue and 1% methylene blue after fixing with glutaraldehyde. Scale bar-200 μm. (**D**) Histogram representing length of total and individual cell processes in TCE treated and control cells. At least 100 cells from each sample in every experiment were counted for process outgrowth analysis. (**E**) Histogram showing average of number of processes in TCE treated and untreated cells. Values are representative of mean ± SEM. ‘*’ (*P*<0.05) and ‘**’ (*p*< 0.01) represent statistical significant difference between control and TCE treated groups.

Anti-proliferative effect of TCE with increasing concentration on these cells was further confirmed by MTT assay. Cells were treated with TCE at concentrations ranging from 10 μg/ml to 1000 μg/ml for 72 h and the cell viability was analyzed. The IC_50_ (concentrations of extract leading to 50% inhibition of cell growth) for C6, U87MG and HeLa cells was about 200 μg/ml and for PC3 cell it was about 500 μg/ml (Figure 2A). Based on these results, 250 μg/ml and 350 μg/ml concentration of TCE was selected for further studies on C6 glioma cells. TCE was further fractionated with hexane, chloroform, ethyl acetate and butanol. Out of these fractions, hexane and chloroform fractions were found to have anti-proliferative property in C6 glioma cells (Figure 2B). Preliminary MTT results showed that IC_50_ value for hexane fraction was approximately 15 μg/ml and for chloroform fraction 20 μg/ml. Bright field images of 1% Toluidine Blue and 1% Methylene Blue stained cells clearly showed highly differentiated morphology of TCE treated cells (Figure 2C). Processes outgrowth data analysis indicated increase in total length of processes by 74.81% in 250 μg/ml and 84.72% in 350 μg/ml TCE treated cells as compared to control cells (Figure 2D). Also there was significant increase in total number of processes in 350 μg/ml TCE treated cells (Figure 2E).

### TCE induced senescence in glioblastoma cells

**Figure 3:**
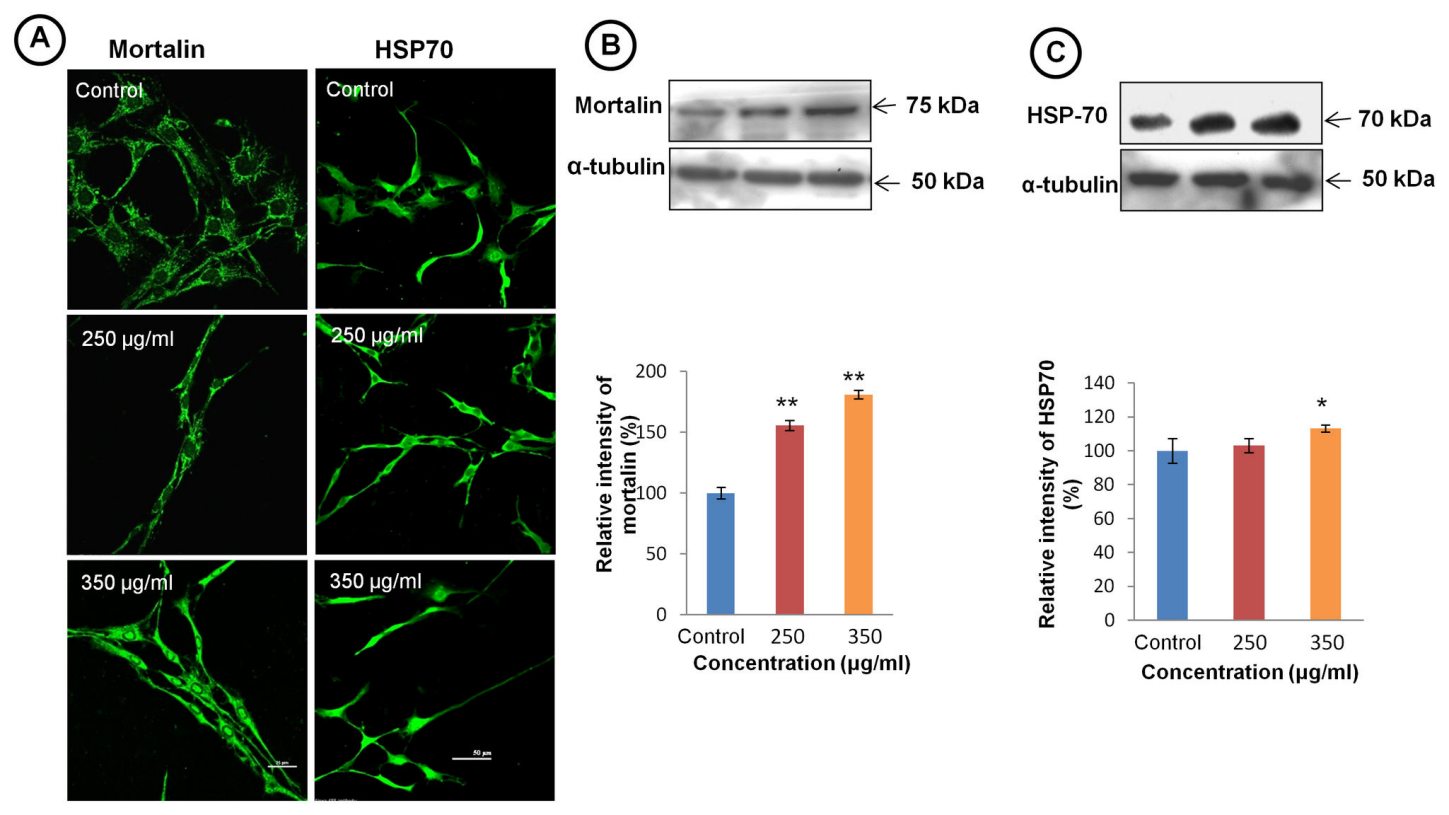
TCE treatment induces senescence in C6 glioma cells. (**A**) Representative confocal images of C6 glioma cells immunostained for mortalin (left panel) showing shift of immunostaining from perinuclear to pancytoplasmic and then to nucleus at higher dose (Scale bar- 25 μm). Immunostaining of C6 cells for HSP 70 (right panel) shows differential expression of HSP70 in TCE treated cells (Scale bar- 50 μm). (**B**) Representative western blot hybridization signals of mortalin. Histogram representing percentage change in mortalin expression in TCE treated and control group. (**C**) Representative western blot hybridization signals of HSP 70 expression. Histogram representing percentage change in expression of HSP 70 in TCE treated and control group. Values are presented as mean ± SEM. ‘*’ (*P*<0.05) and ‘**’ (*p*< 0.01) represent statistical significant difference between control and TCE treated groups.

To explore whether TCE induces senescence in glioma cells, we further examined TCE treated cells with well established senescence marker mortalin, a highly conserved molecular chaperon. Immunostaining data showed pancytoplasmic expression of mortalin in more than 90% of cells in TCE treated group, while in untreated cells most of the staining appeared in perinuclear region (Figure 3A left panel). The overall expression of mortalin was also found to be upregulated in TCE treated cells as compared to control. It was observed that with increasing concentration of TCE nuclear expression of mortalin was also increased and maximum nuclear expression was seen in 350 μg/ml concentrations. Western blot analysis of mortalin also supported the immunostaining data (Figure 3B). Expression of stress response protein HSP70 was also increased significantly in TCE treated cells in comparison to control (Figure 3A right panel). The immunostaining was further supported by western blot hybridization results (Figure 3C). Both mortalin and HSP70 belong to same heat shock protein family and characteristic of cells undergoing senescence. 

### TCE modulated apoptosis and cell survival pathways

**Figure 4:**
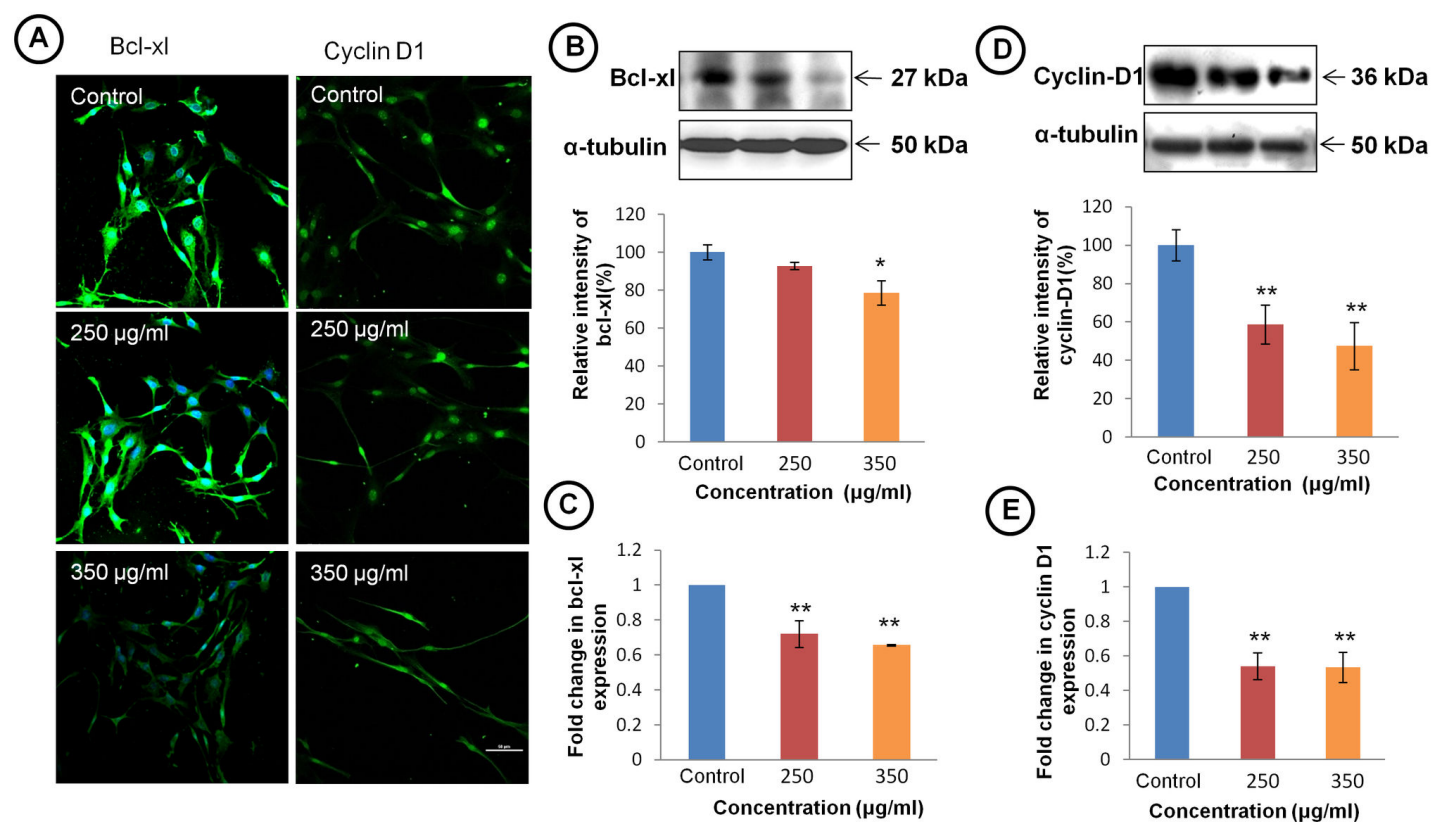
TCE inhibits anti-apoptosis and cell cycle promoting genes. (**A**) Confocal images of immunostaining of bcl-xl (left panel) and cell cycle regulator protein cyclin D1 (right panel) in TCE treated and untreated C6 cells (Scale bar- 50 μm). (**B**) Representative western blot hybridization signals of bcl-xl (upper panel). Histogram (lower panel) representing the relative change in expression of bcl-xl. (**C**) Histograms representing expression of mRNA of bcl-xl in control and treated cells. Gene expression is represented by ΔΔCt value of bcl-xl after normalising with 18S RNA as endogenous control. (**D**) Representative western blot hybridization signals of cyclin D1 in TCE treated and control group (upper panel). Histogram (lower panel) represents relative change in expression of cyclin D1. (**E**) Histograms representing expression of mRNA of cyclin D1 in control and treated cells. Gene expression is represented by ΔΔCt value of cyclin D1 after normalising with 18S RNA as endogenous control. Values are presented as mean ± SEM of at least three independent experiments. ‘*’ (*P*<0.05) and ‘**’ (*p*< 0.01) represent statistical significant difference between control and TCE treated groups.

To elucidate whether TCE modulates signaling pathways associated with apoptosis and cell cycle, we studied the expression of bcl-xl and cyclin D1 in TCE treated cells. The immunostaining of anti-apoptotic gene bcl-xl clearly depicted significant decrease in expression in TCE treated cells (Figure 4A left panel). Western blot and mRNA analysis also supported the immunostaining results (Figure 4B and C). To further test whether TCE effects the expression of cell cycle regulator proteins, cells were immunostained with cyclin D1 (Figure 4A right panel). The results showed dose dependant decrease in expression of cyclin D1 in TCE treated cells. Immunostaining data was further confirmed by western blot and mRNA expression analysis of cyclin D1 (Figure 4D and E). 

**Figure 5:**
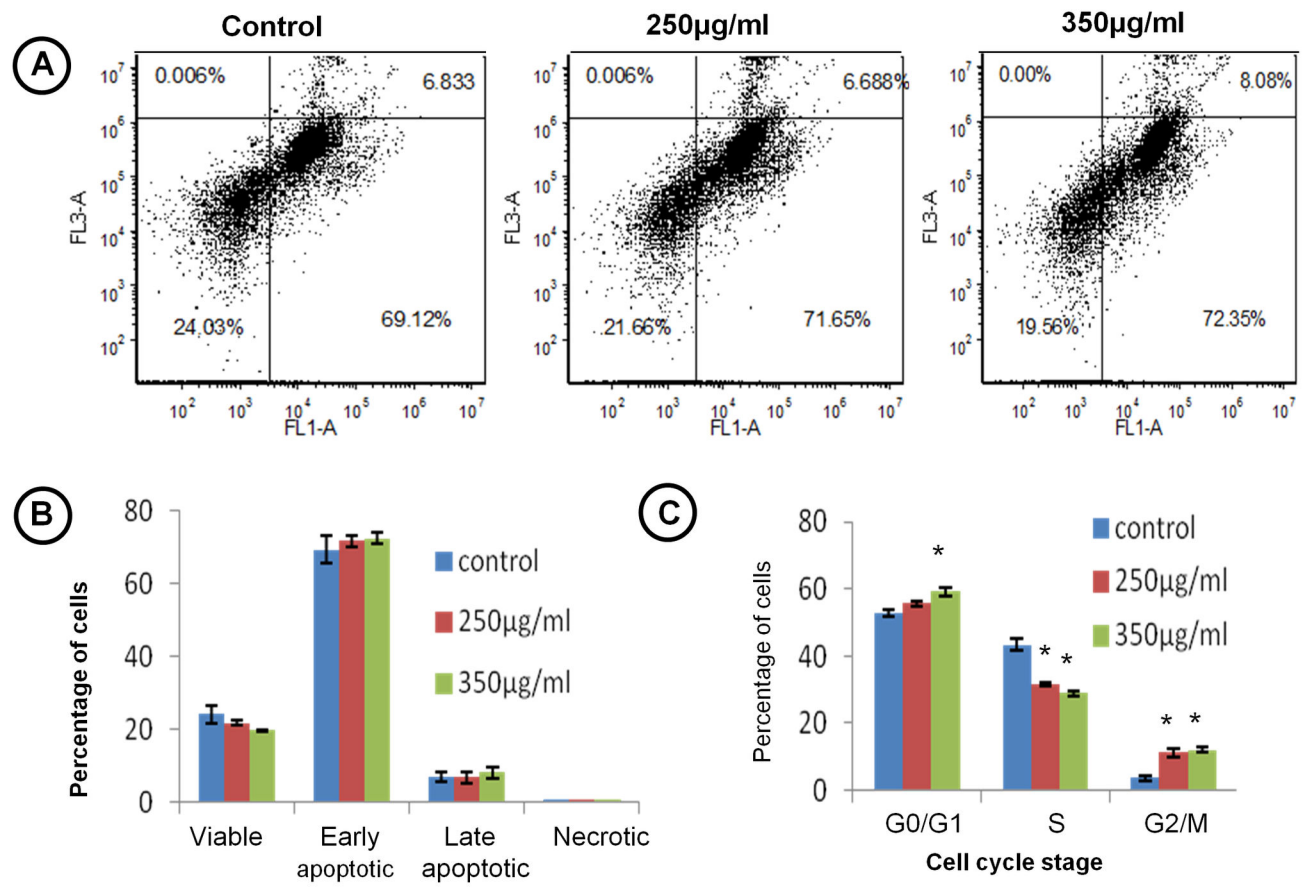
TCE induces apoptosis and cell cycle arrest. (**A**) Distribution of viable, early apoptotic, late apoptotic and necrotic cells analysed by extent of expression of annexin V on the surface of cells and total PI uptake by flow cytometer. (**B**) Histogram showing percentage of cells in viable, early apoptotic, late apoptotic and necrotic stages. (**C**) Histogram representing distribution of cells in G0/G1, S and G2/M phase of cell cycle analysed by PI stain using flow cytometer. ‘*’ represents statistical significant difference (*p*<0.05) between control and TCE treated group.

Apoptosis inducing potential of TCE was also confirmed by Annexin V-FITC and PI staining. Mean value for early apoptotic (72.35%) and late apoptotic (8.08%) cells in TCE treated group were higher than their respective values in control, whereas, viable cell number was reduced (Figure 5A and B) thus deciphering induction of apoptosis in TCE treated cells. Further in the line of above results, cell cycle analysis showed that TCE inhibited cell cycle progression at G0/G1 and G2/M phase as there was a significant increase in the mean percentage of cells in G0/G1 and G2/M phase that was accompanied by remarkable decrease of cells in S phase in TCE treated group (Figure 5C).

### TCE showed anti-migratory potential in C6 glioma cells

**Figure 6:**
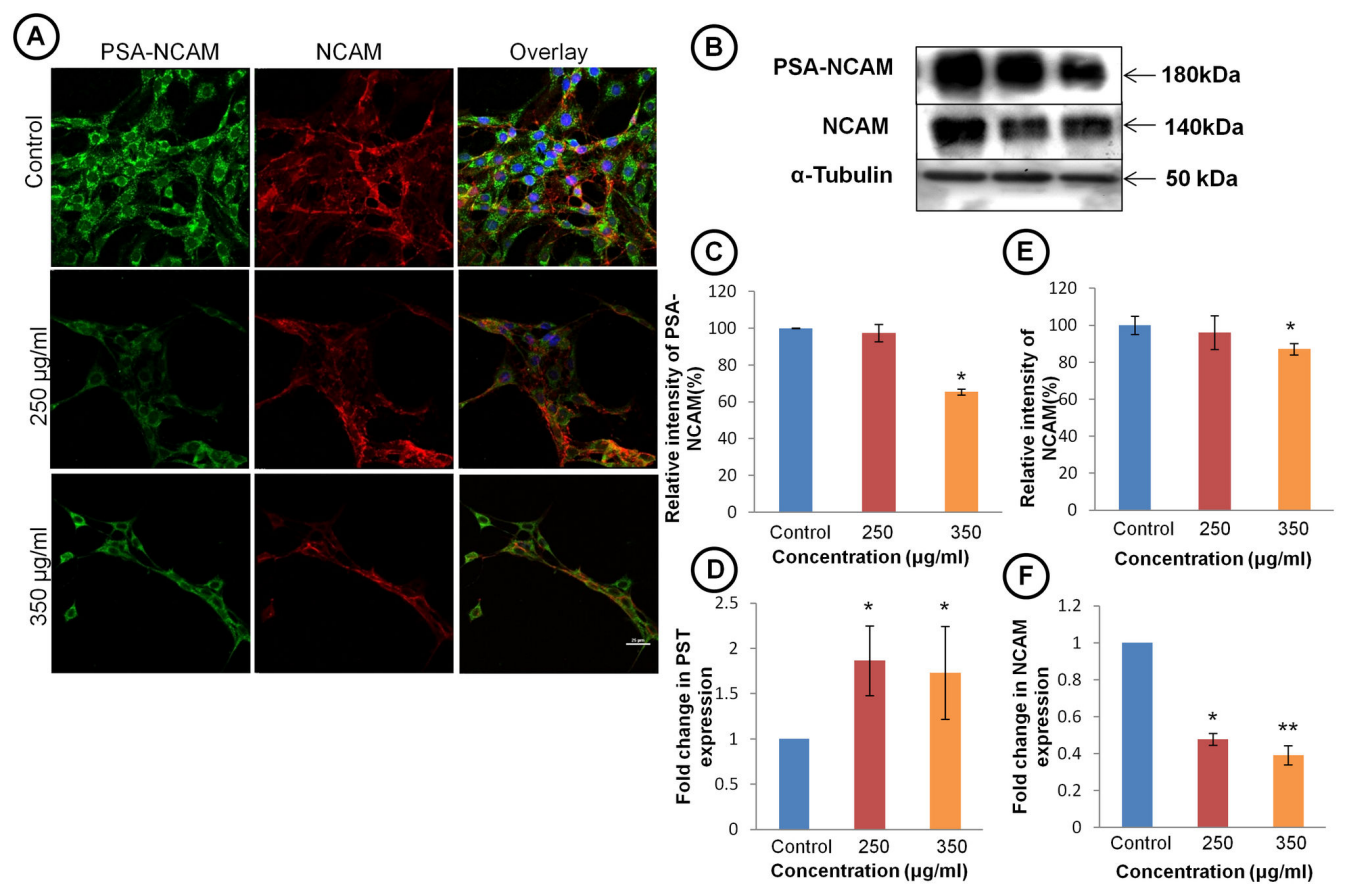
TCE reduces expression of NCAM and PSA-NCAM. (**A**) Immunostaining for PSA-NCAM and NCAM in TCE treated and untreated C6 cells (Scale bar- 25 μm). (**B**) Extent of glycosylation of NCAM estimation by western blot analysis using anti-PSA-NCAM antibody (upper panel). Middle panel represents total NCAM expression. (**C**) Histogram representing percentage change in expression of PSA-NCAM in TCE treated and control cells. (**D**) Histograms representing expression of mRNA of PST (enzyme responsible for polysialylation of NCAM moiety) in control and treated cells. Gene expression is represented by ΔΔCt value of PST after normalising with 18S RNA as endogenous control. (**E**) Histogram presenting densitometric analysis of western blot of NCAM showing decrease in expression of NCAM in dose dependent manner in C6 glioma cells. (**F**) Histograms representing expression of mRNA of NCAM in control and treated cells. Gene expression is represented by ΔΔCt value of NCAM after normalising with 18S RNA as endogenous control. Values are presented as mean ± SEM. ‘*’ (*P*<0.05) and ‘**’ (*p*< 0.01) represent statistical significant difference between control and TCE treated groups.

**Figure 7:**
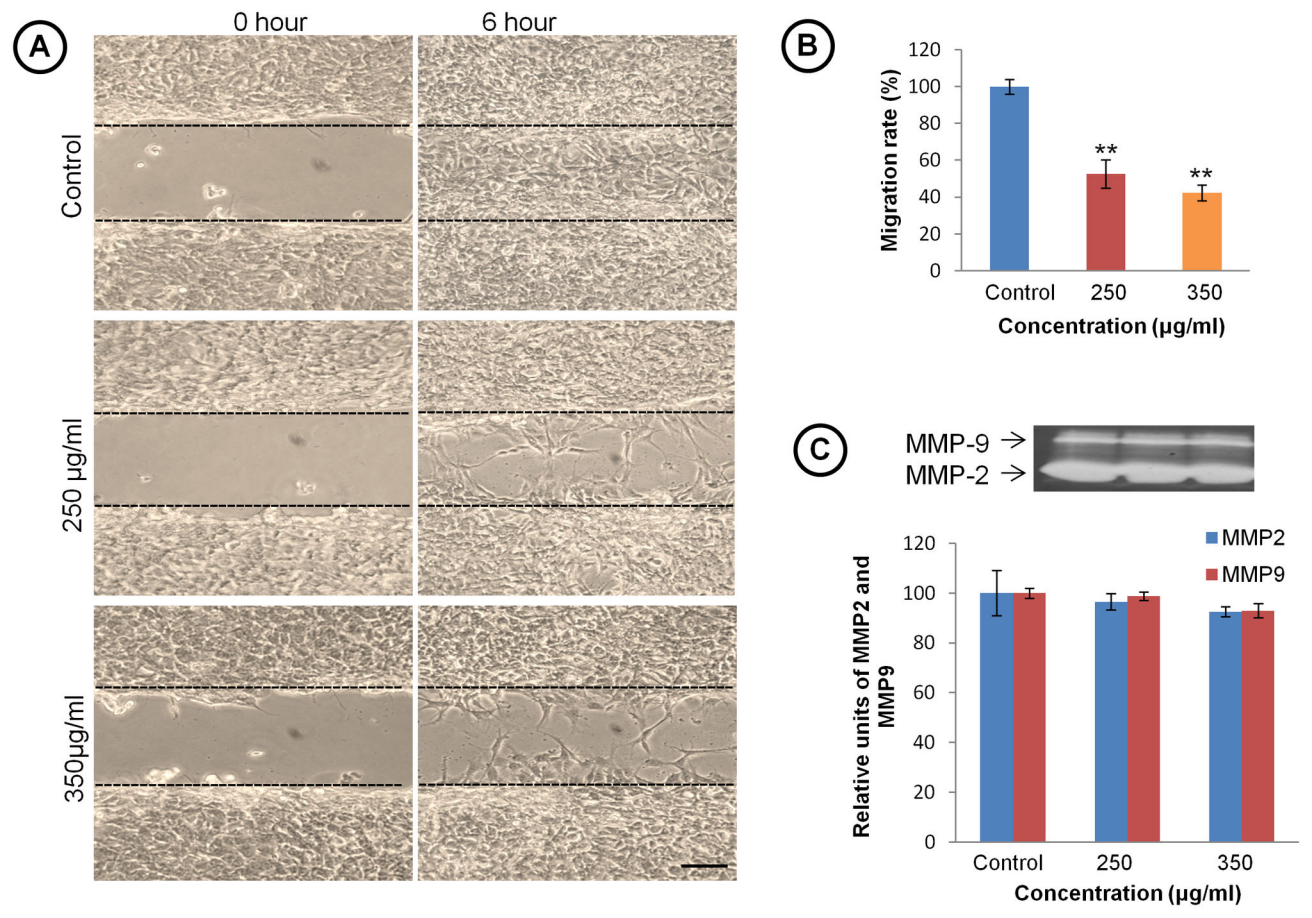
TCE exhibit anti-migratory property in C6 glioma cells. (**A**) Representative Phase contrast images of C6 glioma cells in wound scratch assay to analyze motility of C6 cells. Images show the width of scratch at zero hour and after 6h with and without TCE treatment (Scale bar- 200 μm). (**B**) Histogram representing percentage change in migration rate of C6 glioma cells in TCE treated group. Values are presented as mean± SEM. (**C**) Representative MMP zymogram for control and TCE treated groups. Histogram represents densitometric analysis of MMP bands. . ‘*’ (*P*<0.05) and ‘**’ (*p*< 0.01) represent statistical significance difference between control and TCE treated groups.

Since glioblastoma multiformae are notorious for their highly malignant and invasive properties, we further studied expression of cell adhesion molecule NCAM and its polysialylated form PSA-NCAM in TCE treated and control group (Figure 6A, left panel). Though Immunostaining and immunoblotting data for PSA-NCAM showed significant down regulation at translational level (Figure 6B first panel and C), but quantitative real time PCR data showed increase in expression of polysialyltransferase enzyme (PST) in TCE treated cells (Figure 6D). Immunostaining and western blot data suggested significant decrease in NCAM expression in 350 μg/ml TCE treated group (Figure 6E). Real time quantitative PCR data confirm the down regulation in NCAM expression also at transcriptional level (Figure 6F). To further confirm whether differentiated glioma cells show retardation in their migration rate, we performed wound scratch assay in untreated and TCE treated cells. As shown in Figure 7A, untreated cells were able to invade scratched area and fully recolonize it within 6 h. Treatment with TCE strongly inhibited migration of C6 cells into the scratched area and after 6 h of treatment very few cells were seen to migrate into the scratched area. Quantitative analysis further confirmed a significant decrease (40-53%) in cell migration in C6 cells following TCE treatment (Figure 7B). Further gelatin zymogram analysis was performed to assess the activity of MMP-2 and 9 matrix metalloproteinases to correlate with the anti- migratory property of TCE but no significant change was observed in the MMP-2 and MMP-9 activities (Figure 7C). 

## Discussion

The idea of differentiation therapy by converting malignant cells into normal cells was conceived by G. B. Pearce for terato-carcinoma patients in 1961 [56]. The therapy is based on the development of therapeutic agents that induce terminal differentiation consequent to elimination of cancer cells. Glioblastomas are the most common and highly invasive primary brain tumors. Despite of availability of various radio and chemo-therapies, most of the patients die within one year of diagnosis. In Ayurveda, various medicinal plants have been reported to possess anticancer properties but their mode of action is largely unknown. Using phenotypic information of anticancer compounds used in Ayurveda, Fauzi et al. (2012) predicted ten most enriched targets through *in silico* target prediction method that include primary targets involved in cancer progression such as PTP1B and T-cell Protein Tyrosine Phosphatase (TC-TCP) and synergistic targets such as efflux pumps, P-glycoprotein, opening new avenues for Ayurvedic drug research [57]. *Tinospora cordifolia* is an important component of various ayurvedic decoction used to treat diseases of nervous system and other vital organs like liver, pancreas and kidney. Some previous studies reported immunomodulatory and anticancer properties in water and ethanolic extract of *Tinospora cordifolia* [6,37]. The current study provides first evidence that TCE also possesses antiproliferative, differentiation-inducing and anti-migratory activity in human and rat glioma cells.

Treatment of C6 glioma, U87MG, HeLa, and PC3 cells with TCE (250 and 350 μg/ml) for 72 h significantly reduced their rate of proliferation and Inhibition in proliferation was dose dependent. The IC_50_ value for C6, U87MG and HeLa cells was at 250 μg/ml and for PC3 cells; it was approximately 450 μg/ml, thus suggesting that brain cancer cells are more sensitive to TCE. Fractionation with hexane and chloroform further reduced the effective IC_50_ value to about 6-10% of TCE (^≈^200 μg/ml). Our lab is further continuing work on the identification and characterization of the active components of TCE.

*Tinospora cordifolia* has been shown to have antiproliferative activity in various hepato-carcinoma, lymphoma, and bone cancer cell lines [11,30]. Anti-proliferative property of TCE may be the result of induction of differentiation and senescence as depicted by enhanced expression of GFAP which plays an important role in maintaining the normal astrocyte morphology and growth [58,59]. Upregulation of GFAP expression coupled with morphological changes in C6 cells after TCE treatment may suggest that TCE has differentiation inducing potential. C6 cells transfected with GFAP cDNA showed significantly reduced tumor growth while anti-sense GFAP-transfected astrocytoma cells showed increased invasiveness and growth [60]. Further senescence inducing potential of TCE was confirmed by study of heat shock family proteins, mortalin and HSP70. Mortalin expression in perinuclear spaces in transformed tumor cells and pancytoplasmic in normal cells indicates the activation of senescence pathway in the TCE treated cell. This shift of mortalin expression from perinuclear to pancytoplasmic spaces in transformed cells has been correlated with induction of senescence [40,61]. Consistent with these observations, TCE treated cells showed relocalisation of mortalin from perinuclear spaces to pancytoplasmic space and interestingly, at higher dose there was pronounced expression of mortalin in nucleus, which was not observed in control and 250 μg/ml TCE treated cells. A recent study has suggested that nuclear translocation of mortalin is important for neuroblastoma differentiation, where its interaction with retinoic acid receptors (RAR and RXR) in the nucleus, play important role in RA triggered neuronal differentiation [62]. The enhanced expression of mortalin in nucleus at higher dose of TCE may suggest that mortalin may be an important target for differentiation inducing signaling cascade by TCE. Overexpression of mortalin was also accompanied by up regulation of another stress response protein HSP70. HSP70 is an essential ATP-dependant molecular chaperon and highly involved in neuronal and glial cells differentiation and process outgrowth and it is found to be up regulated in cells undergoing differentiation [63,64].

Further, TCE treated C6 cells showed downregulation of cyclin D1 protein which is required for cell cycle transition from G0/G1 to S phase [65]. It is an important CDK regulatory molecule which plays key role in the translocation of CDK4/CDK6 complex from cytoplasm to nucleus for the progress of G1/S phase transition [66]. Genetic aberration and over expression of cyclin D1 gene have been associated with higher degree of malignancy and increased rate of cell proliferation in several human neoplasms and glioblastomas [67,68]. A recent study reported that in many gliobalstoma, there is suppression of CDKN2A which results into overexpression of cyclin D1 [69], resulting in increased invasive properties of cells and is associated with the enhanced activity of proMMP-2 and MMP-9 [70]. Decrease in cyclin D1 expression in TCE treated cells was followed by arrest of cell cycle progression at G0/G1 and G2/M phase. TCE, being a multi-component system, seems to target multiple cell cycle check points simultaneously, resulting in higher number of cells in G0/G1 and G2/M phase and lesser population in S phase in TCE treated group in comparison to control. Inhibition at G1 phase and reduction in cyclin D1 expression level indicate that glioblastoma cells are undergoing differentiation on TCE treatment. Inhibition of the G1 regulating genes CDK4 or Cyclin D1 in glioblastoma cells may lead to the restoration of the G1 checkpoint and subsequent glial differentiation as the cyclin D1-cdk4 axis is the primary gateway through which mitogenic information is channelled [71]. As cell cycle arrest is a prerequisite of differentiation, it is reasonable to relate the role of TCE in regulating cell cycle and leading to G0/G1 and G2/M cell cycle arrest with down-regulation of cyclin D1 and consequently differentiation of the C6 glioma cells. 

Although glioblastoma are mostly resistant to differentiation and hence apoptosis, but TCE treatment was observed to downregulate the anti-apoptotic gene bcl-xl. Bcl-xl gene is normally over expressed in tumor cells and prevents apoptosis leading to continued cellular proliferation. This gene is also reported to inhibit chemotherapy induced apoptosis [72]. Inhibition of bcl-xl both at transcriptional and translational levels by TCE is in line with findings with other natural products showing anticancer properties, like curcumin, andrographolide, and proanthocyanidines [73,74]. Annexin V-FITC/PI staining study further supported this observation as there was increase in early apoptotic cell population which may be due to the induction of differentiation in C6 glioma cells that led cells to undergo programmed cell death.

Further, the expression of NCAM in TCE treated cells was reduced significantly both at transcriptional and translational levels which was further accompanied by significantly reduced polysialylation over NCAM. The mRNA expression of PST enzyme was found to be increased in TCE treated cells, suggesting that downregulation of NCAM expression itself may be the main cause of reduced glycosylation resulting in lower expression of PSA-NCAM. NCAM is widely expressed during embryogenesis, down-regulated in the course of differentiation to be re-expressed during progression of some tumors [75,76]. Apart from adhesion activity, NCAM moiety is highly involved in GDNF mediated signaling in cell migration and axonal outgrowth and play important role during development and injury [77]. In most of the tumors NCAM along with its polysialylated form is found to be upregualted in tumor cells and polysialylation of NCAM moiety was found to be decisive for its interaction with its ligands and direct tumor growth by controlling its heterophilic interaction [75]. Further upregulated expression of NCAM tumor-derived endothelial cells was found to favor cellular organization into capillary like structure indicating its role in neo-angiogenesis [78]. The reduced expression of NCAM and PSA-NCAM may also be responsible for inhibiting migration of glioma cells independent of MMP-2 and 9 expressions, as there was no repopulation in scratched area in TCE treated cultures. Reduced rate of repopulation of cells in TCE treated cultures in wound scratch assay may be the collective outcome of differentiation, apoptosis and cell cycle arrest which inhibited their migration as metastatic aggressiveness of the tumor is inversely related to its differentiation status. 

Although the use of various compounds like retinoids, taxol, paclitaxel, and PKC inhibitors have been shown to have therapeutic potential but the very nature of glioma exhibiting resistance against chemotherapy and radiotherapy demands new therapeutic drugs [79]. Differentiation inducing effect of TCE was found to be comparable to the effect of ATRA (All Trans-Retinoic Acid) that we have used to induce differentiation in C6 cells in our previous studies [80]. Current data suggests that TCE may have the potential to induce differentiation in C6 glioma cells by targeting different pathways related to cell proliferation, differentiation, senescence and ultimately apoptosis. Further anti-migratory potential seen in cells exposed to TCE may be helpful in controlling metastasis of brain tumors. TCE, being multi-component system, appears to affect multiple pathways for its anti-cancer and differentiation inducing role in C6 glioma cells instead of targeting a single protein or pathway. Although *Tinospora cordifolia* is often recommended in Indian Ayurvedic system of medicine but the mechanistic aspects of its beneficial effects are largely unknown and also potential of its bioactive components is yet to be recognized. Since majority of the reported differentiating agents in glioma (including retinoids) are heat-labile and water insoluble, so the evaluation and characterization of the aqueous ethanolic extract and its active components for discovery of potentially safe glioma-therapeutic phytochemicals is highly warranted. In the light of present data that TCE strongly inhibited proliferation and migration of glioma cells and led cells to undergo differentiation and programmed cell death, it is conceivable that this plant may prove to be a potential candidate for glioblastoma therapy. Our future work will focus on the identification of active components of TCE and search for their potential targets in the multiple pathways observed in the current study.
